# Recursive Filtering for Zero Offset Correction of Diving Depth Time Series with GNU R Package diveMove

**DOI:** 10.1371/journal.pone.0015850

**Published:** 2011-01-28

**Authors:** Sebastián P. Luque, Roland Fried

**Affiliations:** 1 Department of Biological Sciences, University of Manitoba, Winnipeg, Canada; 2 Fakultät Statistik, Technische Universität Dortmund, Dortmund, Germany; Institut Pluridisciplinaire Hubert Curien, France

## Abstract

Zero offset correction of diving depth measured by time-depth recorders is required to remove artifacts arising from temporal changes in accuracy of pressure transducers. Currently used methods for this procedure are in the proprietary software domain, where researchers cannot study it in sufficient detail, so they have little or no control over how their data were changed. GNU R package diveMove implements a procedure in the Free Software domain that consists of recursively smoothing and filtering the input time series using moving quantiles. This paper describes, demonstrates, and evaluates the proposed method by using a “perfect” data set, which is subsequently corrupted to provide input for the proposed procedure. The method is evaluated by comparing the corrected time series to the original, uncorrupted, data set from an Antarctic fur seal (*Arctocephalus gazella* Peters, 1875). The Root Mean Square Error of the corrected data set, relative to the “perfect” data set, was nearly identical to the magnitude of noise introduced into the latter. The method, thus, provides a flexible, reliable, and efficient mechanism to perform zero offset correction for analyses of diving behaviour. We illustrate applications of the method to data sets from four species with large differences in diving behaviour, measured using different sampling protocols and instrument characteristics.

## Introduction

Zero offset correction of depth is one of the first considerations in analyses of diving behaviour data from time-depth recorders (TDRs). Pressure transducers in TDRs often “drift” over time due to temperature changes and other factors, so that recorded depth deviates from actual depth over time at unpredictable rates. Moreover, accuracy and precision vary depending on the instrument, its resolution, and sampling protocol. Therefore, it is crucial to calibrate depth measurements if valid biological interpretations of diving behaviour are to be made.

For diving animals, such as marine mammals and seabirds, the problem of zero offset correction is simplified by the cyclical return to or from the surface as study animals perform their dives throughout the deployment period, thereby providing a reference for calibration. However, this adjustment is typically done via proprietary software provided by instrument manufacturers which implement methods that are, unfortunately, not fully documented for users, so researchers have little or no control over this procedure and cannot know how their data were corrected e.g. [1]–[4].

In this paper, we describe, demonstrate, and evaluate the performance of a method implemented in GNU R [5] package diveMove [6]. We apply this technique to data sets from four species with large differences in diving behaviour, obtained using instruments with different characteristics and sampling protocols to illustrate the general utility of this tool. Our aim is to offer an alternative method for zero offset correction in the Free Software domain that diving behaviour analysts can fully study, control, and modify. The method produces reliable results with a flexible and efficient user interface for controlling the procedure.

## Methods

### Ethics Statement

Research on Antarctic fur seals was carried out in accordance with the ethical guidelines set by Institut Polaire Français Paul Emile Victor (IPEV) for Terres Australes et Antarctiques Françaises (French Antarctic and Austral Territories). The Ministere de l'Agriculture et de la Peche provided a certificate (No. 7200) authorizing research on live animals.

### Description

The method consists of recursively smoothing and filtering the input time series using moving quantiles. It uses a sequence of window widths and quantiles, and starts by filtering the time series using the first window width and quantile in the specified sequences. The second filter is applied to the output of the first one, using the second specified window width and quantile, and so on. In most cases, two steps are sufficient to detect the surface signal in the time series: the first to remove noise as much as possible, and the second to detect the surface level. Depth is corrected by subtracting the output of the last filter from the original.

The method relies on the runquantile function from the caTools package [7], which implements the moving quantile algorithm using a compiled C program, so the procedure is relatively fast. Missing data can be optionally removed before calculating the moving quantiles, if the missing data phases do not involve changes in surface trend.

## Results

### Demonstration

**Figure 1:**
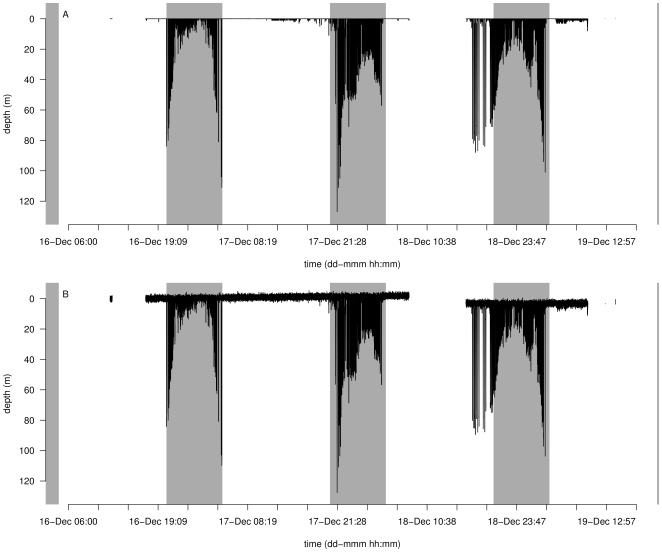
Subset of “perfect” data set to be corrupted and used as reference to measure performance of the “filter” method for zero offset correction (A), and corrupted data set to be used as input (B).

First, we load diveMove and a *TDR* object that is free of any pressure transducer errors, except for a few brief periods that are of no interest and a 2 m offset that we correct for. The object contains two foraging trips performed by an adult female Antarctic fur seal (*Arctocephalus gazella* Peters, 1875), which was equipped with a Wildlife Computers MK8 *TDR* (sampling frequency: 5 s, resolution: ±1%) For the purpose of this demonstration, data from the first foraging trip are enough, so the object is subset to reduce processing time. We introduce a dry period between the second and third day of this subset to simulate a multi-trip TDR record. This data set (Figure 1A) will be used as reference to measure performance of the procedure, and corrupted with noise, drift, and level shift to provide input for the algorithm:

R> library(diveMove)

R> (seal <- readTDR(“ag_mk8_ok.csv”, concurrentCols  = 4:5))

Time-Depth Recorder data — Class TDR object

 Source File: ag_mk8_ok.csv

 Sampling Interval (s): 5

 Number of Samples: 120156

 Sampling Begins: 2001-12-15 16:07:00

 Sampling Ends: 2001-12-22 14:59:55

 Total Duration (d): 6.953

 Measured depth range: [-10, 129]

 Other variables: light temperature

R> d <- getDepth(seal)

R> d[d <2] <- 2

R> seal@depth <- d – 2

R> divesTDRclean <- seal[10001:70000]

R> d <- getDepth(divesTDRclean)

R> d[36000:42000] <- NA

R> divesTDRclean@depth <- d

We add Gaussian noise (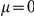 m, and 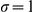 m) to the whole time series, a negative 2-m drift for the first “foraging trip”, and a positive 3-m level shift for the last (Figure 1B). Depth is assumed to increase positively along its scale, following the convention used in most TDR instruments.

R> divesTDRcorrupt <- divesTDRclean

R> d <- getDepth(divesTDRcorrupt)

R> set.seed(1234)

R> epsilon <- rnorm(length(getDepth(divesTDRcorrupt)))

R> d.noise <- d + epsilon

R> divesTDRcorrupt@depth <- d.noise

R> d.drift <- d.noise

R> drift <- seq(0, -2, length.out  =  (36000 - 8000) +1)

R> d.drift[8000:36000] <- d.drift[8000:36000] + drift

R> divesTDRcorrupt@depth <- d.drift

R> d.shift <- getDepth(divesTDRcorrupt)

R> d.shift[36000:60000] <- d.shift[36000:60000] +3

R> divesTDRcorrupt@depth <- d.shift

Different strategies are required to select window width and quantile for the filters at each step, depending on the data. The objective of the first median smoothing step is to remove noise from the surface measurements. The choice should be a compromise between smoothing and avoiding erosion of the surface signal, so this step requires a relatively narrow window. If sampling frequency does not allow for frequent sampling of surface intervals between dives, it is not possible to remove all the noise in the time series without eroding the surface signal (i.e. combining measurements belonging to adjacent dives due to over-smoothing), preventing the next step from properly identifying the surface. Therefore, any remaining noise has to be dealt with in the next step.

Erosion of the surface signal with increasing window width is exacerbated in sections of frequent deep diving with relatively brief inter-dive intervals. To mitigate this problem, the smoothing/filtering process can be limited to observations where the surface is likely to be found, using the argument depth.bounds.

The second filtering step should use a moving window that is as wide as possible, depending on the drift, missing data pattern in the data, and window width of the previous filter. The following strategies are recommended:

- No drift and no level shifts: window width can be as large as the entire data set.
- No drift and level shifts: if the level shifts are separated by missing data, then window width should be limited by the duration of the gaps of missing data to avoid adjacent levels influencing each other. In other words, brief gaps of missing data where a large level shift has occurred would require narrow window widths. The method cannot properly handle cases where different subsequent levels are not separated by missing data, because they require a very narrow window to follow the shift. However, if the shifts are relatively small, a larger threshold for surface measurements can be used during dive analyses to absorb the error due to shifts in these cases.
- Drift and no level shifts: window width should be based on the slope of the drift: steep drifts require smaller window widths.
- Drift and level shifts: a combination of strategies for “no drift and level shifts” cases and “drift and no level shifts”.

These recommendations are, to a large extent, based on the relationship between window width, the length (duration) of missing data sequences, and quantile. The function runquantile maintains a fixed window width throughout the time series, regardless of the number of missing data in each window. Thus, calculation of the quantile is based on 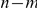 observations, where 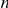 denotes window width and 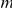 denotes the number of missing observations in any particular window. If all observations within a window are missing, the quantile is also missing. Given these properties, what happens to the quantile when the moving window crosses different levels, and what effect do missing data have on it in these cases?

The answer is that the quantile of the window may be “polluted” by observations from different levels. In the absence of missing data, the smaller of the levels determines the output quantile in case of an upward level shift, until a sufficiently large majority of observations come from the same level as the center of the window. Missing data between different levels have the effect of reducing the number of observations polluting the window. Considering that the moving window is center-aligned, the number of missing data in a window should be larger than 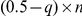, where 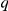 denotes the particular fractions in the interval 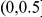 used to calculate the quantile, for the quantile to represent the current “true” local level as good as possible, given 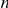. When 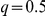 (the median), window width is irrelevant, and the median will represent the local level correctly, whereas at least 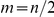 missing values are required to achieve the best possible representation of local level for any quantile. Therefore, when using very small quantiles, window width should not be chosen larger than twice the number of missing values between different levels, in order to get reliable estimates of the surface close to a level shift.

The choice of quantile to extract the surface signal depends on the amount of noise left over from the first step, but should be relatively small. Higher quantiles are required if residual noise is large. If little or negligible noise remains after the first step, a quantile close to the minimum (0.01–0.05) is appropriate. In the corrupted data, noise is relatively large so, after some trial and error, we choose a window width of 12 (1 min) for the first smoothing step (median), and 720 (1 h) for the second, filtering, step. We also bound the process to depths between −6 m and 8 m, where the surface is likely to be found according to Figure 1. Bounding the process within given depth limits means that the moving window filtering mechanism is performed only on the data within those limits. Measurements that are outside these limits are linearly interpolated after each step, so that the output of each filter is as long as the input time series. Similarly, missing data (depth) can be excluded from the filtering process, although they remain missing in the output of the filters. The latter option is appropriate for cases without level shifts, where it helps to speed up the process. Finally, we select quantile 0.35 for the second step, as considerable noise remained after median smoothing.

R> tt <- getTime(divesTDRcorrupt)

R> d <- getDepth(divesTDRcorrupt)

R> K <- c(12, 720)

R> P <- c(0.5, 0.35)

R> d.filter <- diveMove:::.depthFilter(depth  =  d, k  =  K, probs  =  P,

+ depth.bounds  =  c(−6, 8), na.rm  =  FALSE)

**Figure 2:**
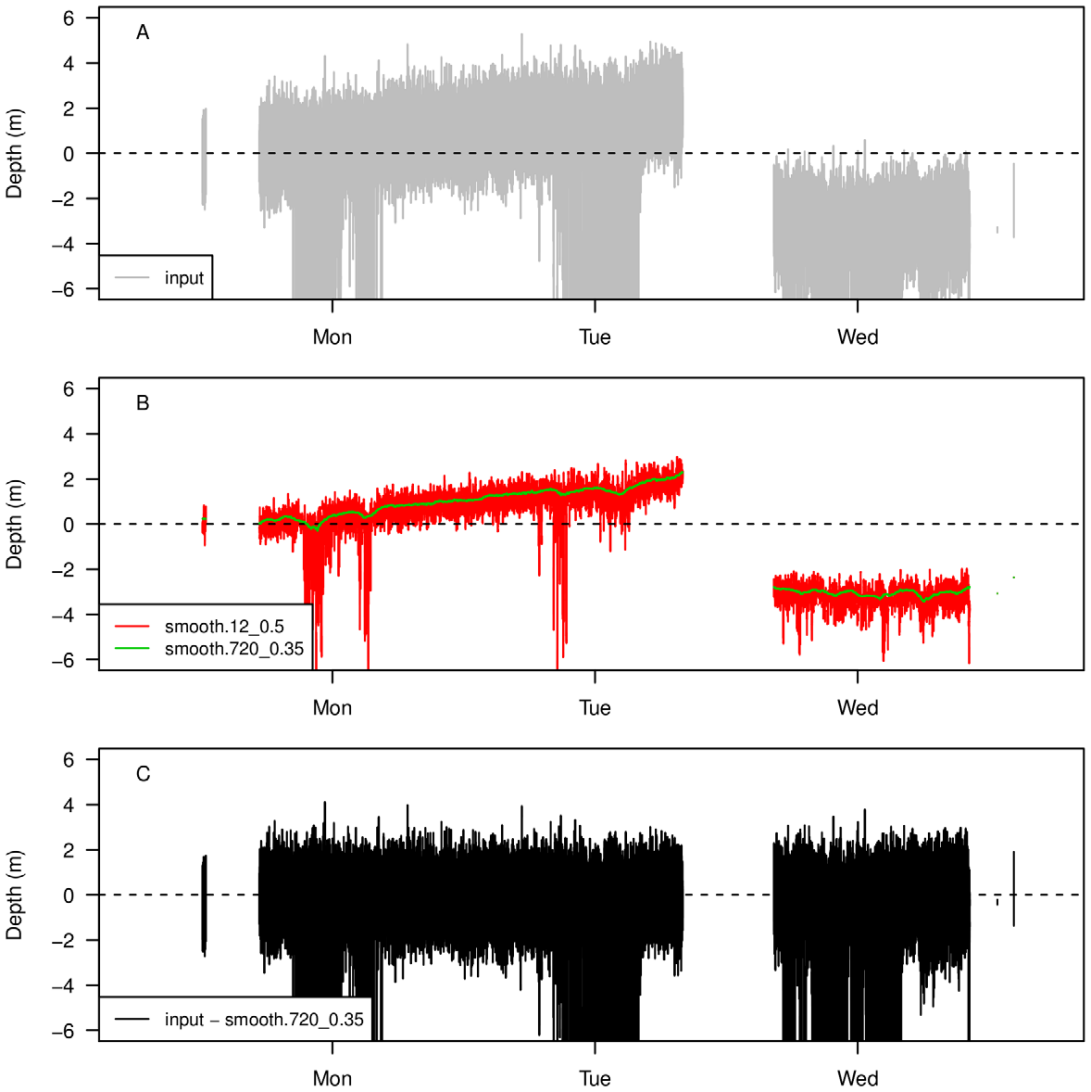
Result of filtering method for zero offset correction of diving depth time series. Corrupted input time series (A), first filter (median) using a moving window of size 12 (1 min) and second filter (0.35 quantile) using moving window of size 720 (1 h) (B), and corrected depth (corrupted series minus last filter) (C). The y-axis limits are restricted to be approximately equal to the range of surface depth in the time series for clarity.

Median smoothing removed approximately 50% of the noise observed at the surface, and the second 0.35 moving quantile followed approximately the center of the range of remaining variation observed at the surface (Figure 2). Therefore, minimum “corrected” depth was below zero (−4.11 m), so cannot be used for further diving behaviour analyses. However, if error is assumed to be symmetrical around zero, and the filtering method bisects the error across zero, then negative depth could be considered error and set to zero for further analyses. This would also require using a dive threshold that is larger than this error.

### Evaluation

**Figure 3:**
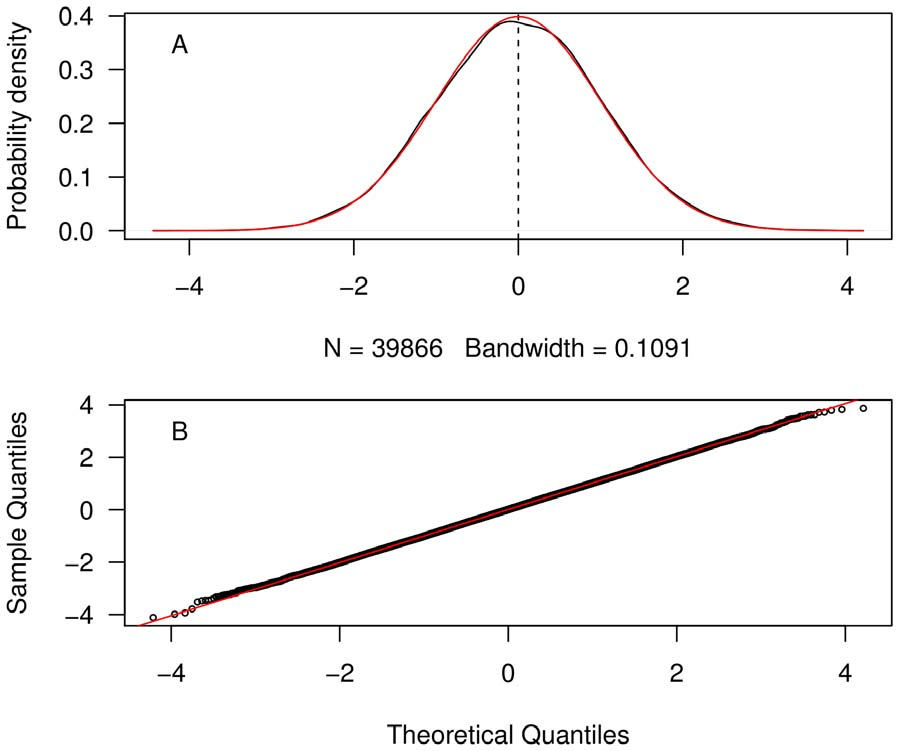
Probability density (A) and “Q-Q” deviations (m) of corrected depth time series from the original, clean, data set (black solid line), and that of normal Gaussian noise (red solid line) introduced into the original time series (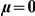 **,** 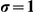 **m) (B).**

To evaluate the performance of the method, we use the Root Mean Square Error (RMSE): 
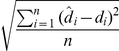
, where 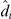 is the 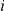-th corrected depth and 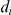 is the 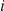-th original, clean, depth. The RMSE was similar to the standard deviation of the Gaussian noise introduced into the time series: 1.01 m. The distribution of deviations (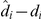) was significantly different from that of the normal noise introduced (Kolmogorov-Smirnov test 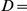 0.007, 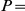 0.03), but the difference was small (Figure 3). The latter could be removed by applying a standard median filtering step to the output.

### Applications

The following applications illustrate the performance of the method with data sets from four species with large differences in diving behaviour. Note that some of the instruments used to record diving depth have been discontinued, and the quality of pressure transducers used in more recent models is superior.

#### Leatherback turtle

**Figure 4:**
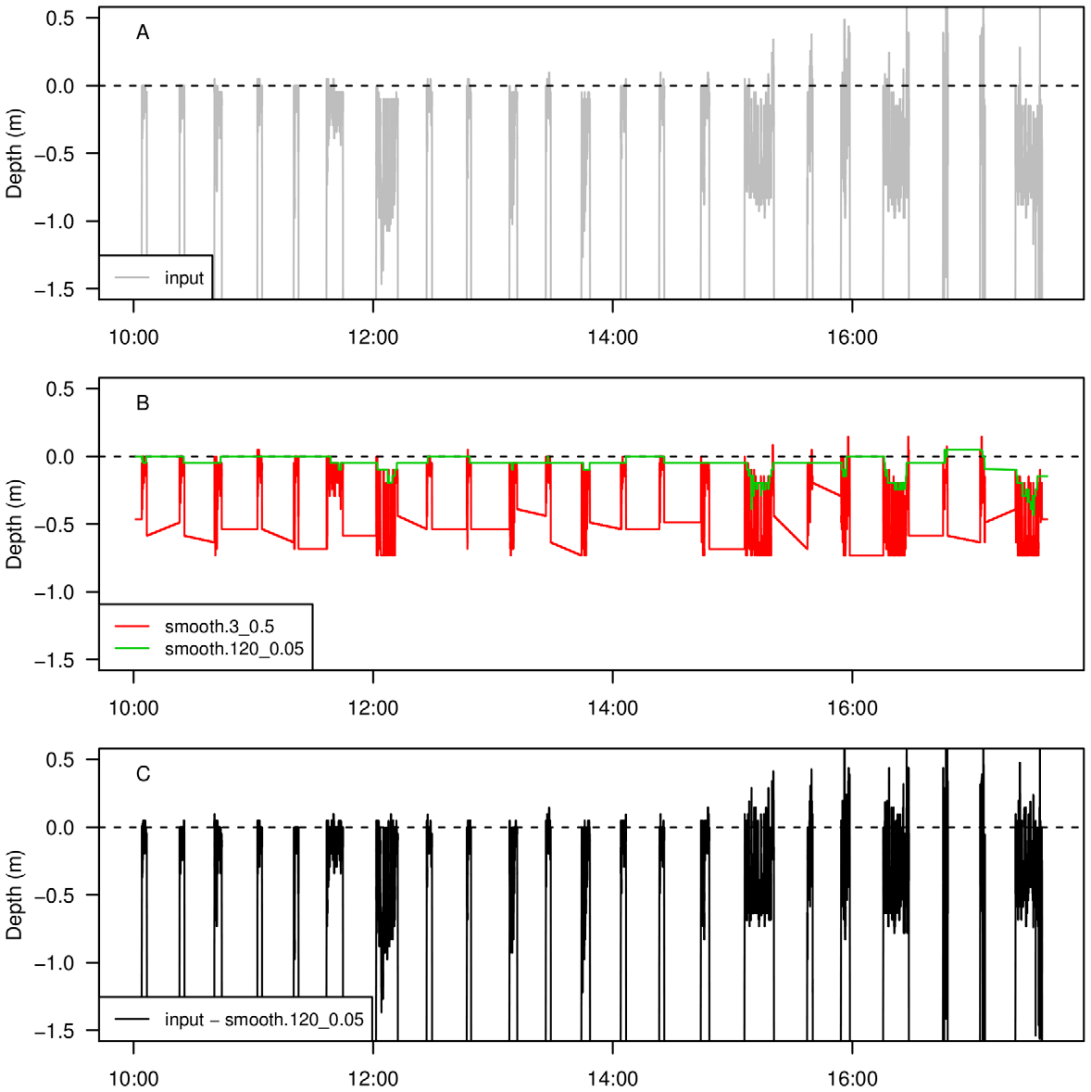
Zero offset correction of TDR data from a leatherback turtle. Input time series (A), first filter (median) using a moving window of size 3 (3 s) and second filter (0.05 quantile) using moving window of size 120 (2 min) (B), and corrected depth (input series minus last filter) (C). The y-axis limits are restricted to be approximately equal to the range of surface depth in the time series for clarity.

Large variation in diving behaviour of leatherback turtles (*Dermochelys coriacea* Vandelli, 1761) has been reported [8]–[10], depending on location and reproductive status. We use previously published data from a leatherback turtle from French Guiana [9] to illustrate the ZOC procedure for divers with subtle diurnal patterns in diving depth, and alternating dives with large differences in shape and depth [9]. The turtle was equipped with a Little Leonardo *TDR* , which sampled depth every 1 s at a resolution of 0.05 m [11] for 10.8 d (see Figure 8a in [9]). However, we focus on a 7.6 h period, where maximum diving depth was 63 m, and data display pressure transducer drifts of approximately 1.75 m. Depth readings less than about −0.4 m, which depart from the overall readings corresponding to the surface, are presumably artifacts caused by high temperatures near the ocean surface (Figure 4A). Therefore, we use a 3 s window width for median smoothing, followed by a 120 s window width for 0.05 quantile filter. We bound the procedure between −0.25 m and 0.75 m, where the surface is found (Figure 4B). The corrected time series displays surface readings from approximately −1.25 m to 0.25 m (Figure 4C).

#### Cassin's auklet

**Figure 5:**
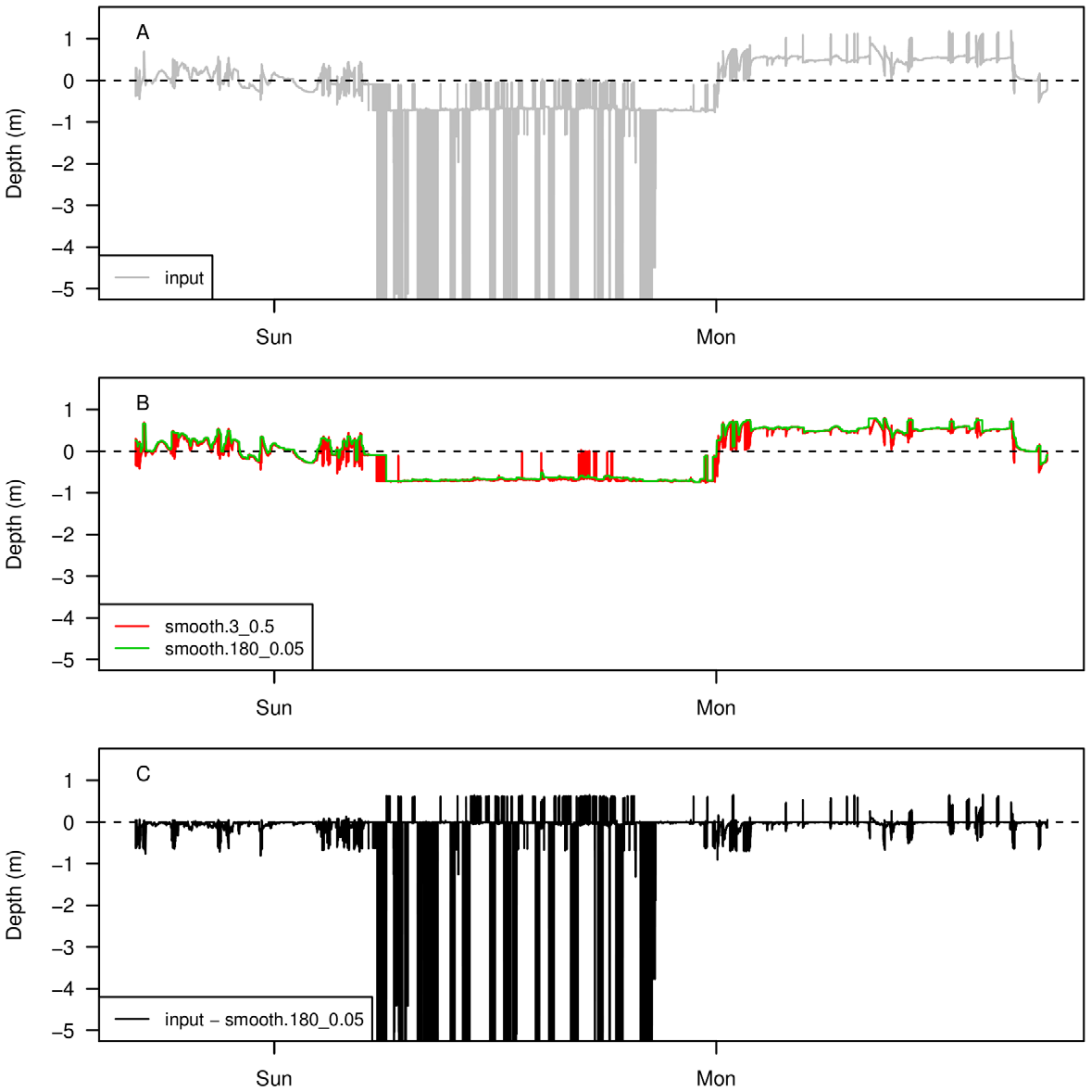
Zero offset correction of TDR data from a Cassin's auklet. Input time series (A), first filter (median) using a moving window of size 3 (9 s) and second filter (0.05 quantile) using moving window of size 180 (9 min) (B), and corrected depth (input series minus last filter) (C). The y-axis limits are restricted to be approximately equal to the range of surface depth in the time series for clarity.

We use data from an incubating Cassin's auklet (*Ptychoramphus aleuticus* Pallas, 1811) from the Pacific coast of British Columbia, to illustrate the performance of the method when diving is relatively shallow (<30 m), brief (<1 min), and frequent (<1 min surface intervals) [12]. The bird was equipped with a Lotek LTD_1100 *TDR* , which sampled depth every 3 s at a resolution of 0.015 m [12] for 2 d. Data recorded by the instrument display level shifts, short-term pressure transducer drifts, and no missing values (Figure 5A). Noise is relatively small (<1 m) in the data, so we choose a 9 s window width for an initial median smoothing step, followed by a 0.05 quantile filter, using a 9 min window width (Figure 5B). These choices did not result in removal of all noise in the data, but they represent the compromise required for smoothing in the first filter, and that required to allow subsequent filters to properly adapt to level shifts and drifts in the absence of missing data. The result is a time series where the surface is found between −0.9 m and 0.9 m (Figure 5C).

#### King penguin

**Figure 6:**
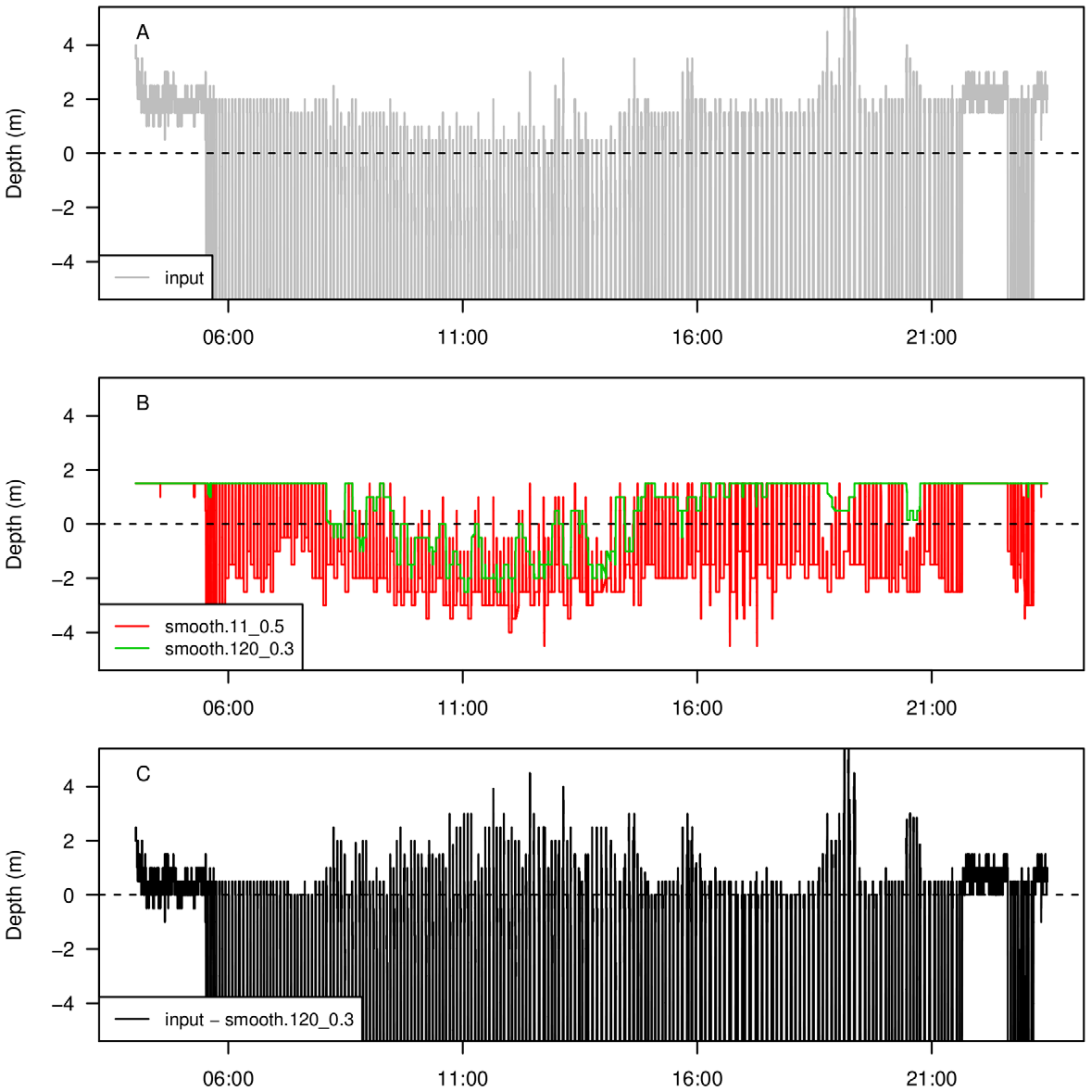
Zero offset correction of TDR data from a king penguin. Input time series (A), first filter (median) using a moving window of size 11 (11 s) and second filter (0.3 quantile) using moving window of size 120 (120 s) (B), and corrected depth (input series minus last filter) (C). The y-axis limits are restricted to be approximately equal to the range of surface depth in the time series for clarity.

Diving behaviour of king penguins displays a diurnal pattern, whereby penguins rarely dive below 40 m during the night, but diving depth may exceed 200 m during the day [13], [14]. We apply our ZOC procedure to data from a king penguin studied at South Georgia (P. N. Trathan and N. Ratcliffe, pers. comm.). The penguin was equipped with a Wildlife Computers MK7 *TDR* (discontinued), which sampled depth every 1 s at a resolution of 0.5 m for 0.8 d. Maximum diving depth was 196 m. Unlike the Cassin's auklet data set, this diving depth time series is characterized by highly unstable pressure transducer performance, where high temperature at the surface is associated with depth readings of less than −2 m (P. N. Trathan and N. Ratcliffe, pers. comm.), and the surface varies from −2 m to 4 m (Figure 6A). Therefore, we use two sequential filters: an initial 11 s window width for median smoothing, followed by a 120 s window width for 0.3 quantile filter, and bound the process to depths between −2 m and 5 m because the surface fluctuates within these limits (Figure 6B). Due to the combination of frequent deep diving and unstable pressure transducer performance near the surface, the quantile in the last filter was chosen to be large enough to smooth the noise in the series remaining after the first filter, yet small enough to detect the surface signal. The output time series displays the surface between −5 m and 3 m (Figure 6C).

#### Short-finned pilot whale

**Figure 7:**
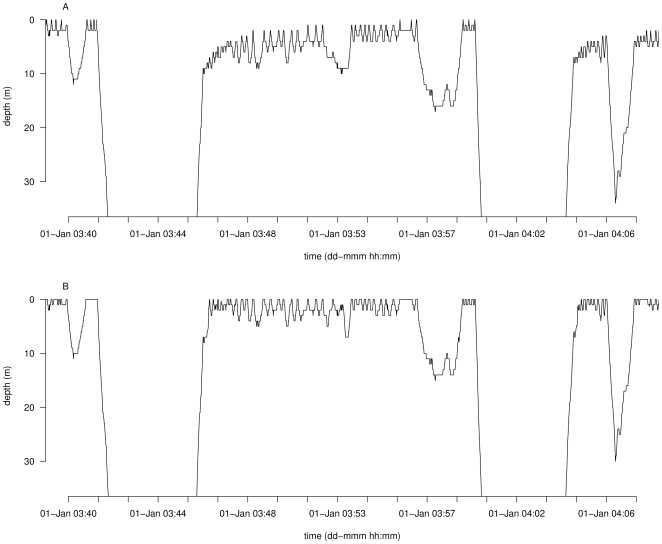
Comparison of ZOC adjusted and unadjusted TDR data from a short-finned pilot whale. Input time series (A), corresponding to period presented in [11], and corrected depth (B). The y-axis limits are restricted to the top 35 m of this period for clarity. Date and time on x-axis were set arbitrarily because input consists of seconds.

Short-finned pilot whales (*Globicephala macrorhynchus* Gray, 1846) are fast and deep divers, which may dive down to 1000 m below the surface for about 20 min [15]. We use data from a short-finned pilot whale studied off Hokkaido, Japan (Baird and Amano, unpublished data in [11]) to illustrate the ZOC procedure for divers with these characteristics. The dolphin was equipped with a Wildlife Computers MK6 *TDR* (discontinued), which sampled depth every 1 s at a resolution of 1 m [11] for about 6 h. Maximum diving depth was 237 m. Data recorded by the instrument display short-term pressure transducer drifts, mostly due to temperature-related effects on sensor performance (see Figure 1 in [11]), known as thermal hysteresis. These drifts induce short-term level shifts during diving bouts with brief surface intervals because the pressure transducer cannot adapt to the rapid changes in temperature, and they blend into the noise at the surface, which is relatively large (>3 m) (Figure 7A).

Given the characteristics of this time series, therefore, we use three sequential filters: an initial median smoothing filter with 7 s window width, followed by a 0.1 quantile filter with 7 s window width, ending with a 0.01 quantile filter with 30 s window width. We also bound the process to depths between 0 m and 10 m because the surface fluctuates within these limits. Due to the thermal hysteresis in depth readings, it is not possible to simply use two filters, with the second consisting of a wide window width to reduce noise. Therefore, the filtering choices represent a compromise between smoothing in the first two filters, and allowing the last filter to adapt to the hysteresis and related short-term drifts in the time series. The result is a time series where the surface is found between 0 m and 8 m (Figure 7B).

## Discussion

### Availability and Future Directions

Package diveMove is available from the main GNU R's CRAN repository (http://www.r-project.org or its mirrors. The latest development version is always available from R-Forge (http://r-forge.r-project.org/projects/divemove).

Despite its flexibility, reliability, and efficiency, the recursive filtering and smoothing method implemented in diveMove requires user-defined window widths and quantiles. Future development efforts will focus on automating these choices, which might be guided by the goal of minimizing deviance of depth measurements from zero for purported dives that are within the nominal resolution of the instrument. Further sophistication may be achieved using data-adaptive window widths so that filter outputs follow surface signal more closely.
